# Conjugates of Ultrasmall Quantum Dots and Acridine Derivatives as Prospective Nanoprobes for Intracellular Investigations

**DOI:** 10.3390/nano11092160

**Published:** 2021-08-24

**Authors:** Pavel Linkov, Pavel Samokhvalov, Maria Baryshnikova, Marie Laronze-Cochard, Janos Sapi, Alexander Karaulov, Igor Nabiev

**Affiliations:** 1 Laboratory of Nano-Bioengineering, National Research Nuclear University MEPhI (Moscow Engineering Physics Institute), 31 Kashirskoe Highway, 115409 Moscow, Russia; palinkov@mephi.ru (P.L.); p.samokhvalov@gmail.com (P.S.); ma_ba@mail.ru (M.B.); 2 Laboratoire de Recherche en Nanosciences, LRN-EA4682, Université de Reims Champagne-Ardenne, 51 Rue Cognacq Jay, 51100 Reims, France; 3 Laboratory of Experimental Diagnostics and Biotherapy of Cancer, N.N. Blokhin Russian Cancer Research Center, 24 Kashirskoe Highway, 115478 Moscow, Russia; 4 Institut de Chimie Moléculaire de Reims, Université de Reims Champagne-Ardenne, 51 Rue Cognacq Jay, 51100 Reims, France; marie.cochard@univ-reims.fr (M.L.-C.); janos.sapi@univ-reims.fr (J.S.); 5 Laboratory of Immunopathology, Department of Clinical Immunology and Allergology Sechenov First Moscow State Medical University (Sechenov University), 8-2 Trubetskaya Str., 119991 Moscow, Russia; drkaraulov@mail.ru

**Keywords:** quantum dots, multifunctional nanoprobes, acridine derivatives, G-quadruplex

## Abstract

Designing nanoprobes in which quantum dots (QDs) are used as photoluminescent labels is an especially promising line of research due to their possible medical applications ranging from disease diagnosis to drug delivery. In spite of the significant progress made in designing such nanoprobes, the properties of their individual components, i.e., photoluminescent QDs, vectorization moieties, and pharmacological agents, still require further optimization to enhance the efficiency of diagnostic or therapeutic procedures. Here, we have developed a method of engineering compact multifunctional nanoprobes based on functional components with optimized properties: bright photoluminescence of CdSe/ZnS (core/shell) QDs, a compact and effective antitumor agent (an acridine derivative), and direct conjugation of the components via electrostatic interaction, which provides a final hydrodynamic diameter of nanoprobes smaller than 15 nm. Due to the possibility of conjugating various biomolecules with hydroxyl and carboxyl moieties to QDs, the method represents a versatile approach to the biomarker-recognizing molecule imaging of the delivery of the active substance as part of compact nanoprobes.

## 1. Introduction

The incidence rate of malignant tumors is continuously growing. During the past decade, the onset of cancer has been detected in about 6 × 10^6^ people per year throughout the world [1,2]. One of the most advanced potential strategies in cancer treatment is the targeted delivery of antitumor agents to cancer cells, which ensures low concentrations of toxic drugs in healthy tissues [3,4,5]. In this case, in addition to the highly specific targeting molecule, the drug may be conjugated with a label, usually a magnetic, radioactive, or photoluminescent one. Optical imaging, which is relatively inexpensive and easy to implement, is currently the leading trend in imaging-guided therapy. It relies on the detection of photons transmitted through tissues and provides noninvasive tracking of drug carriers and monitoring of treatment. Conjugation of an imaging (photoluminescence (PL)) tag to a capture molecule and the drug makes it possible to track the drug delivery in real-time mode [6,7]. Although organic fluorophores (PL dyes and proteins) traditionally used for engineering diagnostic or therapeutic probes have some advantages, such as a high PL quantum yield (QY) and good compatibility with aqueous media, there are also significant drawbacks that limit their use, notably a low photostability and a narrow absorption spectrum, precluding the excitation of the PL of different organic fluorophores at a single wavelength.

Quantum dots (QDs) are nanometer-sized photoluminescent semiconductor nanocrystals (NCs) with a wide spectral range of PL [8]. The QD energy structure and hence light absorption and emission characteristics are largely determined by their physical size due to the quantum confinement effect, so that, along with uniquely bright PL, QDs are characterized by a narrow PL band whose spectral position is controlled by the NC size [9]. For example, common QDs with a cadmium selenide core can be used as a basis of materials intensely luminescing in the visible region of the spectrum (450–650 nm). Core/shell QDs currently constitute the most widely used QD type. Their photoluminescent (typically CdSe) core is epitaxially coated with a thin shell of a semiconductor with a wider-band gap (e.g., ZnS or CdS) in order to enhance charge confinement in the QD core and shield the core from the outer medium [10,11,12]. The application of the shell also makes it considerably easier to functionalize the QD surface with hydrophilic groups facilitating the transfer of QDs into an aqueous medium, as well as with capture and drug molecules, which is necessary for obtaining multifunctional nanoprobes.

QD-based systems have numerous potential practical applications, which has stimulated efforts towards the development and investigation of QD-based materials and control of their morphology at the nanoscale [13]. The use of QDs as PL tags is typical in their recent application in life science and medicine, where their unique optical characteristics provide numerous advantages over the commonly used organic fluorophores.

The use of QDs as PL labels in biology has been limited by the low colloidal stability of QDs in aqueous medium, non-homogeneity of QD ensemble properties, and strong quenching of PL with the polar water molecules [14]. Optimization of QD characteristics is aimed at ensuring particle uniformity, narrow and symmetrical emission bands, a high PL quantum efficiency, and minimal blinking of individual QDs. The influence of the ligand layer and crystal lattice defects of core/shell QDs on their optical characteristics, crystal growth, etc. has become a major concern of the research community [13]. In terms of chemical synthesis, the latest advances have been made in modifying passivated inorganic shells by using novel shell structures and approaches to the shell growth, which has led to a significant improvement of QD properties. Nevertheless, applications in biomedicine research suffer from the lack of easy, versatile, and reproducible production techniques to prepare highly stable QDs and small physical size for unhindered entry into healthy and cancer cells.

The unlimited growth of tumor cells is usually triggered by factors stimulating cell proliferation. Antitumor drugs either cause cell death or suppress cell proliferation. The search for anticancer therapy focuses on inhibitors of the enzymes involved in DNA replication [15]. Compounds consisting of heterocyclic groups and aromatic cycles with N, S, or O as heteroatoms exhibit a wide range of pharmacological effects. In particular, bioactive heterocyclic substances containing nitrogen attract much interest because of their important roles in DNA transcription, repair, and the cell division machinery, including DNA replication and chromosomes segregation during the anaphase [16,17,18]. Inhibited expression of enzyme-encoding genes by stabilizing DNA may initiate intracellular signals leading to apoptosis of the cancer cell. Ensuring the targeted delivery of pharmaceutical agents into the cell is a crucial issue in the development of anticancer drugs of the new generation [19]. Since cellular membrane defense mechanisms limit drug penetration into cells [20], the use of membrane vectors (molecules with affinity for cell membranes) for transporting pharmaceutical agents is a promising method whose efficiency has been demonstrated for a number of functionally active substances. A properly selected vector can transport the drug molecule or nanoparticle into the cell cytoplasm or nucleus [3]. Various natural polyamines (putrescine, spermine, spermidine, etc.) are convenient vectors for introducing cytotoxic agents into cancer cells [21,22]. The main way to obtaining probes for targeted drug delivery is to modify the pharmacological agent with the vector residue. Additional conjugation of the pharmacological agent with an imaging fluorescence agent [23] ensures both delivery of the active substance into a cancer cell and its spatiotemporal tracking.

One of the current strategies of the development of advanced cancer treatment methods involves targeted drug delivery to tumor cells, which ensures low drug concentrations in healthy tissues. In this case, in addition to the highly specific targeting molecule, the drug can be conjugated with a luminescent label, e.g., a QD. Linking a QD to the targeting molecule and the drug makes it possible to track the drug delivery in the real-time mode. However, the use of QDs as labels is limited by a relatively large hydrodynamic size compared to cell pores, non-homogeneity of QD ensemble properties, and quenching of luminescence by the polar water molecules.

We have developed QDs with advanced colloidal and optical properties for biomedical applications, such as the engineering of compact conjugates of QDs with acridine derivatives, potential anticancer agents capable of effectively stabilizing the telomeric G-quadruplex sequences of human DNA. For this purpose, we (1) synthesized homogeneous defect-free QD cores; (2) coated them with ZnS shells in a controlled manner; (3) synthesized novel DNA ligands and evaluated their biocompatibility; (4) assembled multifunctional nanoprobes and tested them in vitro. The developed method of nanoprobe engineering is versatile because the ligand-coated QD surface allows conjugating not only with acridine derivatives, but also with others pharmacological agents, using carbodiimide coupling chemistry.

## 2. Materials and Methods

### 2.1. Materials

Cadmium oxide (powder, 99.5%), 2-ethyl hexanoic acid (2-EHA, 99%), oleic acid (90%), tri-*n*-octylamine (TOA, 98%), 1-octadecene (ODE, technical grade, 90%), oleylamine (OLA, technical grade, 70%), selenium (powder, 100 mesh, 99.5%), zinc oxide (powder, 99.99% trace metals basis), thiourea (≥99.0%), tri-*n*-octylphosphine (TOP, technical grade, 97%), DL-cysteine hydrochloride (98%), and methylene glycol dimethyl ether (TEGDME, ReagentPlus, 99%) were from Sigma-Aldrich. *n*-hexadecylphosphonic acid (HDPA, 97%), was from PlasmaChem GmbH; the HS-(CH_2_)_11_-PEG_6_-COOH and HS-(CH_2_)_11_-PEG_6_-OH PEG derivatives were from ProChimia Surfaces, Sopot, Poland. The chemicals were used as received without additional purification.

Anhydrous solvents (methanol, chloroform, dichloromethane, hexane, dioxane, toluene, acetone, methyl acetate) were from commercial suppliers (Sigma-Aldrich, Burlington, MA, US). They were used as received or distilled under sodium or CaCl_2_ and stored over 4 Å molecular sieves. All reactions were performed in oven-dried glassware under an atmosphere of nitrogen or argon.

### 2.2. Characterization

The UV–Vis spectra were measured by means of a Carry 60 UV-Vis absorption spectrophotometer (Agilent Technologies, Santa Clara, CA, USA). For recording the PL spectra, an Agilent Carry Eclipse spectrofluorimeter (Agilent Technologies, Santa Clara, CA, USA) was used. The QD hydrodynamic diameter and ζ-potential in a diluted aqueous solution at a low ionic strength were determined from dynamic light scattering using a Zetasizer Nano ZS instrument (Malvern Panalytical Ltd., Worcestershire, UK). The morphology and physical sizes of the QDs were studied using a JEOL JEM 2100 F transmission electron microscope (200 kV; the STEM and HRTEM modes), JEOL Europe SAS, Paris, France. For recording ^1^H NMR spectra at 298 K, we used a Bruker Avance III-600 spectrometer (600 MHz) equipped with a cryoprobe, where CD_3_OD was used as a solvent and TMS served as an internal standard (Bruker France S.A.S, Champs sur Marne, France). 

The mass spectra were measured by means of a Micromass Q-TOF micro-instrument (Manchester, UK), which was equipped with a pneumatically assisted electrospray (Z-spray) source of ions operated in the positive mode (HR ESI-MS). The concentrations of QDs, acridine derivatives, and their conjugates in solutions were determined by spectrophotometry using Beer’s law and the experimentally estimated extinction coefficients of CdSe/ZnS QDs (ε = 50,000 L × mol^−1^ × cm^−1^ at 463 nm) and acridine derivative (ε = 14,000 L × mol^−1^ × cm^−1^ at 264 nm). The extinction coefficients of QDs and acridine derivative were determined by measuring the absorbance spectra of an aliquot of the reaction solution with a known concentration of CdSe/ZnS QDs and the solution with a known mass concentration of acridine derivative, respectively. Gel permeation chromatography of water-soluble QDs and QD–acridine conjugates was performed using Sephadex G100 (particle size, 4–120 μm). Dextran beads were pre-swollen in a phosphate buffer solution (pH 7.2, 50 mM) as a stationary phase. The same solution was subsequently used as the mobile phase. A disposable chromatographic column (6 mm × 60 mm) was filled with pre-swollen Sephadex G100 suspended in the buffer solution (approximately vol, 7 mL). The sample aliquot was placed onto the top of the resin using a pipette. Sample fractions were collected into 2-mL plastic test tubes, the volume of each eluted fraction was 40–70 µL. The fractograms for each component of the samples studied and QD-to-acridine ratios were extracted from the absorbance spectra of each collected fraction using the characteristic absorption bands and molar extinction coefficients of the AL and QDs according to Beer’s law.

### 2.3. Synthesis of CdSe/ZnS Core/Shell QDs

The CdSe QDs were synthesized in accordance with a procedure reported earlier, based on the hot-injection approach [13]. In the synthesis of CdSe cores, cadmium *n*-hexadecylphosphonate and tri-*n*-octylphosphine selenide (TOPSe) were used as precursors, and TOA served as a stabilizing co-agent. The cadmium precursor was obtained in two steps: at the first step, a solution of cadmium 2-ethylhexanoate was obtained by dissolving 1 mmol of cadmium oxide in 2.5 mmol of 2-EHA and 2 mL of ODE under fast heating to 300 °C in argon atmosphere. This was followed by replacement of the 2-EHA residue with the n-hexadecylphosphonate one by adding 150 mg of HDPA, 2 mL of TOA and 6 mL of ODE. Evacuation at 10 mbar for 30 min was followed by slow heating of the solution to 240 °C and exposure under an argon atmosphere for 1 h.

Then the selenium precursor, prepared in a separate vessel by dissolving 2 mmol of Se powder in 2 mL of TOP, was rapidly injected into the solution of the cadmium precursor at 240 °C under vigorous stirring at 1100 rpm. The reaction was stopped after 20 s by intense cooling caused by transferring the reaction mixture into liquid nitrogen. The obtained cores were isolated by precipitation from the crude solution using a twofold volume of methyl acetate, after which the mixture was centrifuged, and the supernatant was removed. The QD pellet was dissolved in 4 mL of toluene and purified by gel permeation chromatography (GPC). GPC was performed in a chromatography column 1 cm in diameter, packed with ~10 g of SX-1 cross-linked polystyrene beads (BIO-RAD) serving as a stationary carrier phase, which were pre-swelled in toluene. Toluene was used as an eluent. The passage of the QD band through the stationary phase was monitored visually, and the product was collected in a single fraction, with the fronts of the QD band eluted from the column being disposed.

After that, the HDPA residues on the surface of the cores were replaced with oleylamine (OLA) in the presence of sodium borohydride [24]. This reagent helped us to remove the tightly bound phosphonic acid residues from the core surface and made it possible for the new OLA ligands with a much weaker affinity for CdSe to passivate it. This was followed by coagulation of QDs with methanol and redispersion of them in toluene. The core solution in toluene was filtered through a 25–50 mm glass filter, mixed with 6 mL of ODE and 6 mL of OLA, and then toluene was evaporated under vacuum.

The 0.61 M zinc stock solution was obtained by dissolving zinc oxide in a 2.05-fold excess of 2-EHA diluted with the appropriate amount of ODE. For obtaining the 0.85 M sulfur stock, thiourea was dissolved in methylene glycol dimethyl ether (TEGDME) by sonication.

For precise deposition of the desired amount of shell monolayers, we estimated the amount of CdSe core NCs from the absorption spectrum of an aliquot of the reaction mixture using the molar extinction coefficient of 2 × 10^5^ l × mol^−1^cm^−1^ taken from the data reported in [25]. A three-monolayer ZnS shell coating was obtained using the successive ion layer adsorption and reaction (SILAR) procedure in the thermocycling mode, with a slight reduction of precursor quantities according to [26]. The precursor injection and shell growth were performed at 120 °C with subsequent heating to 175 °C for the reaction to proceed [27]. The first cycle began with injection of zinc. Before each injection of the sulfur precursor into the reaction mixture, 0.5 mmol of TOP was added to stabilize sulfur-terminated QDs synthesized in the next cycle upon thiourea injection. The shell growth was finalized with an additional injection of the amount of the zinc precursor sufficient for the formation of half of the shell monolayer.

When the shell was completely grown, the procedure described earlier [28] was used to isolate the resultant QDs from the crude solution.

### 2.4. Preparation of Water-Soluble QDs

The preparation of water-soluble QDs was performed as described elsewhere [29], with some modifications. To do this, 5 mg of the QDs was twice purified from the excess of ligands by the dispersion/coagulation procedure using chloroform/methyl acetate, respectively. Then, the QD solution in chloroform was titrated with 2 equivalents of 10 g/L DL-cysteine solution in methanol. After centrifugation, the precipitate was washed twice with methanol by means of dispersion of the aggregated NCs in an ultrasonic bath followed by centrifugation after each washing. Cys-QDs were dissolved in a solution of 50 μL of 0.1 M NaOH and 0.6 mL of MilliQ water. After a brief distillation of residual methanol in a centrifugal concentrator, the QD solution was centrifuged at 10,000 rpm to remove insoluble QDs. At the second stage, DL-cysteine was exchanged with a four-fold excess of polyethylene glycol derivatives (HS-(CH_2_)_11_-PEG_6_-COOH; -OH) with terminal hydroxyl or carboxyl groups at a ratio of 9:1, respectively.

The PEGylated QDs were left overnight for incubation and preliminarily cleaned by centrifugation using Amicon Ultra-15 filter units with a 10 kDa cut-off (Millipore Corporation, Burlington, MA, USA) in 50 mM phosphate buffer (pH 8.0) at 3500 rpm and then purified from excess ligands using GPC in Sephadex G-100 columns according to the protocol developed earlier [28].

### 2.5. Conjugation of QDs with Acridine Ligand

**Figure 1:**
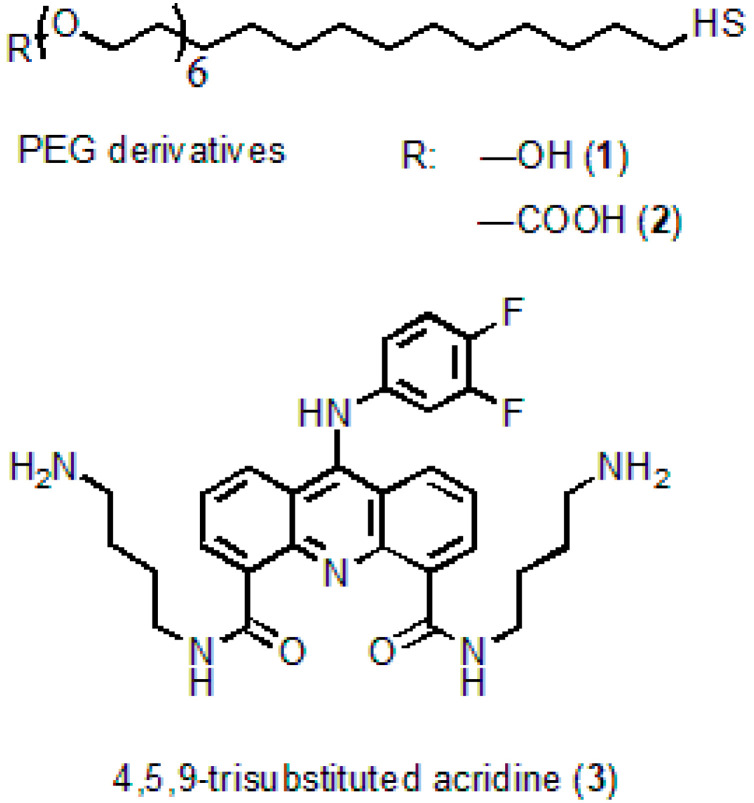
Molecular structures of the polyethyleneglycol (PEG) derivatives and acridine ligand.

*N4,N5*-*bis*(4-aminobutyl)-9-(3,4-difluorophenylamino)acridine-4,5-dicarboxamide serving as an acridine ligand (AL) (Figure 1) was obtained from acridine via seven successive reactions. The molecular structure of the substance was confirmed by ^1^H NMR analysis and mass spectrometry [28]. The procedure of AL synthesis will be described in detail elsewhere.

The QDs and AL were electrostatically coupled by mixing 4 nmol (3 mg) of QDs in the form of a 0.05 M QD solution in 50 mM phosphate buffer (pH 7.2) with a five-fold molar excess of AL. The resultant crude mixture was purified using SEC in Sephadex G100 (Sigma-Aldrich, Burlington, MA, USA).

### 2.6. Cell Uptake

Human monocytes were isolated using the ficoll reagent. Ficoll was placed into a test tube, and fresh human whole blood was added to the top layer of it. The blood was centrifuged at 1500 rpm for 25 min. The layer of mononuclear cells was removed into a separate test tube. Monocytes were washed with PBS containing heparin at a concentration of 5000 U/mL, and the test tube was centrifuged at 1500 rpm for 15 min. The top layer was removed with a pipette. The precipitate was shaken and washed in 10 mL of RPMI-1640 medium at 1000 rpm for 8 min. The monocytes were counted, and 5 × 10^6^ cells per Petri dish were seeded in 1% RPMI-1640 medium supplemented with 2% of human serum. The Petri dishes were placed into an incubator for 1.5 h. Unattached cells were removed with a pipette, and the Petri dish containing monocytes was gently washed with PBS. Afterwards, 20 mL of 1% RPMI-1640 medium (containing 2% of human serum) was placed into the Petri dish.

To analyze the interaction of water-soluble QDs-PEG-OH with monocytes, the QDs in the form of a 1 mg/mL solution were added to the cell medium to obtain the desired concentration. The mixture was incubated at 37 °C for 10 min, after which the uptake of the QDs by human monocytes was studied by means of laser scanning confocal microscopy.

## 3. Results and Discussion

### 3.1. Core/Shell QD Synthesis

An advanced technique for synthesizing ultrasmall monodisperse CdSe QD cores was developed. The cores were obtained by the hot-injection method in the low-polar high-boiling organic solvent 1-octadecene. The CdSe cores were obtained from cadmium *n*-hexadecylphosphonate and selenium tri-*n*-octylphosphine serving as precursors and tri-*n*-octylamine serving as a co-surfactant. This procedure relies on the combination of fast termination of core formation and growth by almost instant cooling of the reaction mixture with liquid nitrogen and multistep high-performance core purification using gel penetration chromatography. A typical high-temperature synthesis of CdSe NCs using the hot-injection procedure yields relatively large NCs (>2 nm) during the first minute of the reaction. In addition, the methods commonly used for termination of CdSe NC growth, such as cooling of the reactor with an air flow or liquid bath, result in an additional increase in the mean NC diameter in the ensemble during a relatively slow cooling. Specifically, for ultrafast stopping of the reaction, we transferred the reaction mixture into liquid nitrogen 20 s after selenium precursor injection. After the evaporation of liquid nitrogen and subsequent melting of the reaction mixture at room temperature, we obtained a stable colloidal solution of ultrasmall CdSe QDs (~1.8 nm in diameter, as determined from the absorbance spectrum using reference data from [25]; Supplementary Figure S1) containing a large number of unreacted precursors. These precursors, had they been present in the reaction mixture at the stage of shell deposition, would have increased the size distribution of the QD ensemble and interfered with the growth of a uniform shell, causing unpredicted spectral shifts in the absorbance and PL spectra of the final QDs. As in all common synthetic procedures, CdSe cores were first separated from the crude nonpolar reaction solution by precipitation using methyl acetate as a coagulant. However, this method has a disadvantage: the efficiency of the purification from the precursor depends on the size of the isolated cores. This is because smaller cores have a relatively high resistance to coagulation, even if the medium contains a considerable excess of the polar precipitant. On the other hand, the relatively low-polar cadmium precursor in the form of cadmium hexadecylphosphonate can precipitate from the solution upon addition of a large excess of coagulant. Thus, during the isolation of small CdSe cores, simultaneous precipitation of both CdSe NCs and cadmium precursors takes place.

To achieve complete removal of the unreacted cadmium precursor from the CdSe core solution and avoid the aforementioned undesired effects, an additional purification stage was introduced to the synthesis routine. Specifically, we employed GPC with Bio-Beads SX-1 cross-linked polystyrene beads as a stationary carrier and toluene as an eluent. This method, with QDs rapidly migrating through the stationary phase and the low-molecular-weight precursors retained in the carrier pores, has been proven to be efficient for the removal of excess surface ligands or unreacted Cd precursors from CdSe NC ensembles [30].

The GPC purification did not have a strong effect on the position of the first excitonic transition of purified CdSe NCs in the absorption spectra compared to that of the original CdSe cores, which evidenced that the ensemble-average diameter of core NCs remained unchanged. 

The growth of shells on the QD cores relies on two general processes: adsorption of the metal and chalcogen precursors on the NC surface and their subsequent reaction yielding the shell material. The phosphonate anions, commonly employed as capping agents in QD synthesis, can effectively prevent the adsorption of the shell precursors [31]. Thus, to improve the quality of the passivating shell, phosphonate anions on the CdSe core surface should be preliminarily replaced with ligands that have a smaller affinity for the core surface and a higher mobility. Here, we used OLA molecules as such ligands. The ligand exchange was performed through addition of excess OLA to the solution of CdSe cores in toluene in the presence of NaBH_4_, treatment for 15 min under ambient conditions and mild stirring, and subsequent purification by coagulation and centrifugation. The presence of a reducing agent (sodium borohydride), which intensely reduces anions on the QD surface, thereby facilitating their desorption and replacement with OLA molecules [23], substantially enhances the PL of the solution of CdSe cores and ultimately improves the optical characteristics of the core/shell QDs.

Core/shell QDs are typically significantly less homogeneous in size than the original cores, even if shell growth is conducted carefully by dropwise addition of the shell precursors. The layer-by-layer shell growth method allows an increased accuracy of the core/shell QD size distribution in comparison with the classical one-solution approach. However, even the SILAR approach can result in the formation of non-uniform and irregular shells [27].

Here, the epitaxial shell growth was carried out using a SILAR-like approach based on sequential injection of small discrete portions of metal and chalcogen precursors, starting from the metal (in the order metal → chalcogen → metal, etc.) into the reaction mixture. This approach, compared to the classical method involving the continuous injection of two precursors or their mixture using a syringe pump, allows synthesizing QDs with well-controlled structure and shell thickness. Unlike in the classical SILAR method, we designed the injection sequence in such a way that only a half of a monolayer grows during each cycle of injection of a precursor to fully terminate the existing metal or chalcogen atoms on the NC surface with a counter-ion. This allowed us to minimize the coexistence of two precursors in the reactor, and hence homogenous nucleation of the shell-phase NCs. This approach makes it possible to obtain high-quality shells, with the PL QY significantly increased.

**Figure 2:**
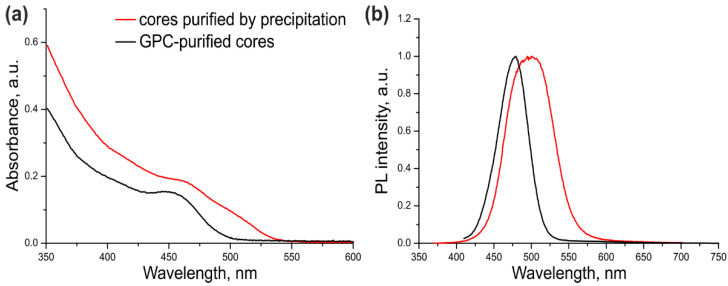
The absorption (**a**) and fluorescence (**b**) spectra of the CdSe/ZnS QDs purified by GPC. The black curves correspond to QDs with GPC-purified cores; the red curves correspond to QDs with cores purified by the common precipitation/redispersion technique.

The optical parameters of the CdSe/ZnS QDs with CdSe cores additionally purified or not purified by GPC were compared in order to estimate the ultimate effect of this procedure on the quality of the resulting core/shell QDs. For this purpose, a three-monolayer ZnS shell was grown on the cores that were purified by dispersion/coagulation alone (Table 1, Sample #1) or by dispersion/coagulation and subsequent GPC (Table 1, Sample #2). The optical properties of both samples are presented in Figure 2.

As seen from Figure 2 and Table 1, the excitonic transition in Sample #1 was “blurred” because of considerable variation of the QD shell thickness in the ensemble, as evidenced by a large PL FWHM. In addition, the PL maximum was shifted towards the red region by 35 nm compared with the PL maximum of the core NCs (Supplementary Figure S1). The cadmium precursors left in the CdSe core solution after incomplete purification formed an inner layer of CdS and/or more complex substances with the general composition Cd_x_Zn_1–x_S in some of QD shells in Sample #1. Sample #2 of CdSe/ZnS QDs was substantially more homogeneous, as demonstrated by its considerably narrower PL band (by a factor of more than 1.5). The red shift of the PL band caused by shell deposition was also shorter (13 nm). These data indicate that the optical characteristics of the QDs with the cores additionally purified by GPC were considerably improved. The PL QY of ultrasmall QDs was measured to be 68% using Coumarin 102 as a reference dye. This PL QY value is far from the maximum possible one, which is typical of such small QDs because of the high probability of PL quenching via various mechanisms.

The average diameter of QDs from Sample #2 estimated from HRTEM (Supplementary Figure S2) was determined to be 4.2 ± 0.7 nm.

To conclude, ultrasmall CdSe core QDs 1.8 nm in diameter were obtained by means of optimized colloidal hot injection synthesis modified with the fast termination of QD growth using liquid nitrogen. Size exclusion chromatography as a method of high-performance purification of the QD cores has been shown to prevent the subsequent non-uniform growth of epitaxial shells with varying composition and to ensure the formation of a QD ensemble with highly homogeneous optical properties. The deposition of a ZnS shell onto the obtained cores resulted in a PL QY increase from 4% to 68%.

### 3.2. Water Solubilization of Ultrasmall QDs

The method of phase transfer of organic-phase-soluble QDs was based on the procedure reported by [32,33] with some modifications.

We performed an adaptation of the method via the precise calculation of the necessary ligand quantities using the data on the composition of the final shell layer obtained using a QD growth model and applying soft solubilization conditions to avoid QD aggregation. Therefore, we estimated the necessary quantity of cysteine for complete passivation of the surface of the ultrasmall core/shell QDs. In the given case, this amount was found to be 319 molecules of the ligand per QD. The minimum diameter and narrow size distribution of water-soluble Cys-QDs were achieved by using a twofold excess of cysteine over the calculated quantity, which is explained by the necessity of ligand excess in the competitive ligand exchange on the QD surface.

At the second stage, we replaced DL-cysteine with PEG derivatives containing terminal hydroxyl or carboxyl groups (HS-(CH_2_)_11_-PEG_6_-COOH; -OH) at a ratio of 9:1, respectively. The hydroxyl derivatives provide chemical inertness in biological media, and the carboxyl derivatives provide a small ζ-potential, better colloidal stability of the QD solution, and the possibility of QD conjugation with biomolecules and enhancement of cellular uptake. Then, we used the size difference between the free ligands and QDs to separate them by means of SEC. Sephadex cross-linked dextran gel beads with different porosities are the most popular stationary carrier phases used in SEC. However, the selection of the porosity value is difficult because the manufacturer’s recommendations on the separating ability are given for globular proteins rather than nanoparticles. Sephadex G25 exhibited a low efficiency of separation of QDs and free PEG derivatives, whereas Sephadex G200 had the best separation efficiency but required too much eluent. The separation quality of G100 was similar to that of G200, and its relatively low porosity provided rapid elution. Thus, it had the best balance between the separation efficiency and elution time. Therefore, it was used to purify the QD samples from unbound PEG ligands and, afterwards, unbound AL.

### 3.3. Characterization of Water-SolubleQDs 

The main characteristics (size distribution and ζ-potential) of the ultrasmall QD solution were estimated using DLS.

Using the adapted approach, we managed to obtain water-soluble QDs that had an average hydrodynamic diameter of 11.7 ± 3.8 nm. The ζ-potential was close to neutral and was determined to be −12.6 ± 6.2 mV, which demonstrated an insignificant charge due to a small amount of negatively charged carboxylic derivatives of PEG. The combination of a stable ligand shell and a slight negative surface charge provides high colloidal stability for more than six months. The variation of the optical density of QD colloidal solution, ζ-potential, and hydrodynamic diameter after six months of storage did not exceed 10%. 

### 3.4. Engineering of Multifunctional Nanoprobes

To make a biomolecule suitable for intracellular imaging, the synthesized molecule should be conjugated to a PL QD without disturbing the biological function of this molecule. The water-soluble QDs stabilized with PEG-COOH were originally planned to be covalently bound with AL containing terminal amino groups by means of carbodiimide coupling, because this is one of the most extensively used strategies for oriented molecule–molecule or molecule–QD conjugation. Covalent methods of conjugation involve the use of commercially available linkers, such as EDC and NHS, to conjugate carboxylic functional groups of ligands to the amine group of QDs or vice versa. The structure of AL was so designed as to provide conjugation with QDs.

However, we found that QDs and acridine derivatives can form complexes even without a crosslinker in a neutral medium (0.1 M phosphate buffer solution, pH 7–8), which was detected due to an instantaneous change in the QD PL intensity when the two components were mixed. The spontaneous formation of electrostatic complexes occurred instantaneously, whereas carbodiimide coupling with the EDC or NHS zero-length crosslinker by the coupling methodology took 2 h. Hence, although the covalent coupling may be more thermodynamically favorable than the electrostatic one, there is no reason to use it for conjugation, because the difference between the reaction rates is too large.

**Figure 3:**
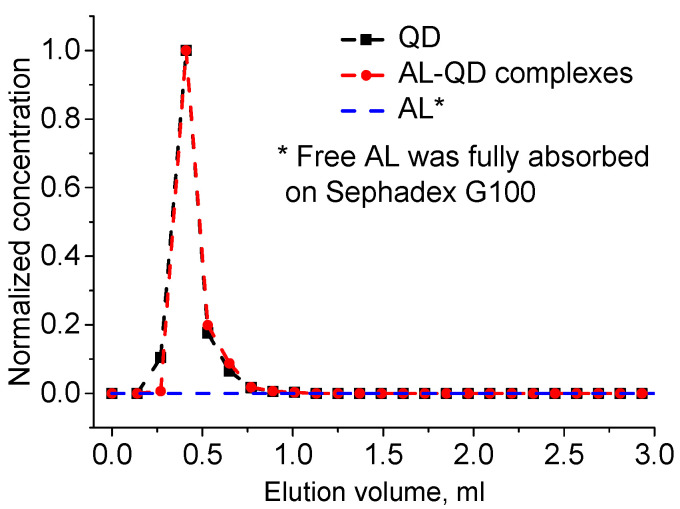
Elution chromatogram of acridine ligand (AL)–QD complexes and their components. GPC with Sephadex G100 as the stationary phase.

The obtained electrostatic complexes were purified from unbound AL molecules by means of GPC using Sephadex G100 as the stationary carrier. The chromatogram of GPC purification of AL–QD electrostatic complexes using Sephadex G100 as the stationary carrier obtained by processing the absorbance spectra of individual fractions is shown in Figure 3. As seen from the figure, the original QDs and their electrostatic complexes with acridine are eluted similarly to pure QDs, as a narrow band, whereas AL is completely retained in the stationary phase, as evidenced by stable coloration of Sephadex G100 when it was passed through the column in the control experiment. In order to estimate the efficiency of AL–QD binding, we compared the absorption spectra of the electrostatic complexes before and after GPC purification. The results showed that the ratio between the characteristic absorption maxima of AL and QDs in the spectrum of the complex was not changed in the course of purification. Thus, the binding resulted in the formation of highly stable electrostatic complexes.

**Figure 4:**
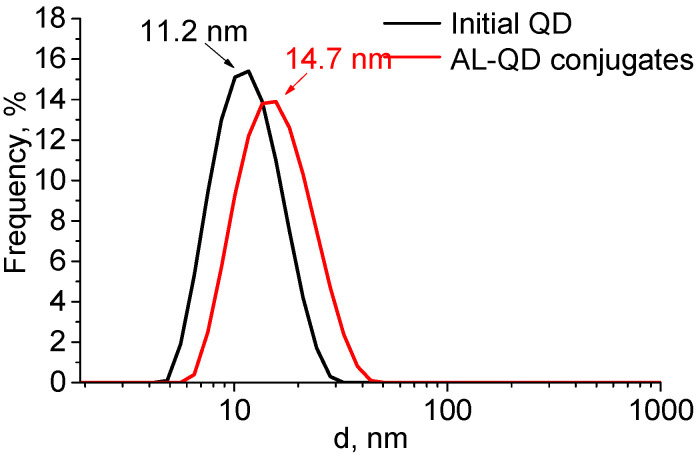
Size distribution of the original CdSe/ZnS QDs and AL–QD complexes in phosphate buffer (pH 8, 0.1 M).

The formation of complexes caused a minor increase in the QD hydrodynamic diameter, from 11.2 to 14.7 nm. The size distribution pattern remained the same, which indicated the absence of aggregation and a highly homogeneous distribution of the ligands over the QD ensemble (Figure 4). The ζ-potential of the QDs increased from −12.6 to −8.6 mV after AL binding. This could be explained by the change in the composition of the QD surface ligands. In addition to the carboxyl groups, which imparted a weak negative charge to the QDs, acridine molecules appeared on the surface. They contained seven nitrogen atoms each, three of which were readily protonated (the primary amine groups of putrescine and the heteroatom in the acridine backbone). This decreased the negative charge of the QD surface, because the positive charges of the amine groups compensated for it.

The PL properties of the conjugates play an important role in their biological applications. Acridine-based ligands, being polyaromatic compounds, constitute an effective system of charge carrier transfer. The HOMO and LUMO energy levels of 4,5-acridine derivatives are comparable with these energy levels of semiconductor NCs [23,34,35], which allows these derivatives to be used as hole-transporting materials in designing photovoltaic [35] and optoelectronic devices [36]. On the other hand, this charge transfer leads to the problem of quenching of the QD PL [37,38] due to photoinduced electron transfer (PET) [39]. For instance, PL QY of the conjugates with a QD-to-AL ratio of 1:5 is reduced twofold compared to the initial QDs. We have found that the QD shell structure is a crucial factor of photoinduced electron transfer from the QDs to the AL, which leads to QD PL quenching. The degree of quenching is mainly determined by the shell structure, with the effect of the QD size being negligible. This prevents acridine derivatives from being used as targeting agents in designing QD-based nanoprobes. Overcoming this problem by using shell structure engineering was addressed in our previous publication [40].

### 3.5. Toxicity and Cellular Uptake of Nanoprobes and Components

An evaluation of the QD toxicity and understanding its mechanisms are prerequisites for the development of biological and medical applications of QDs for imaging, diagnosis, and treatment. However, it is hardly possible to compare the results of the available published studies and draw unambiguous conclusions from them, because these studies used different QDs, cell lines, and analytical techniques. The methods of QD synthesis and modifications of their surface also strongly affect their physicochemical characteristics, and hence their interaction with the cell membrane and cell uptake.

**Figure 5:**
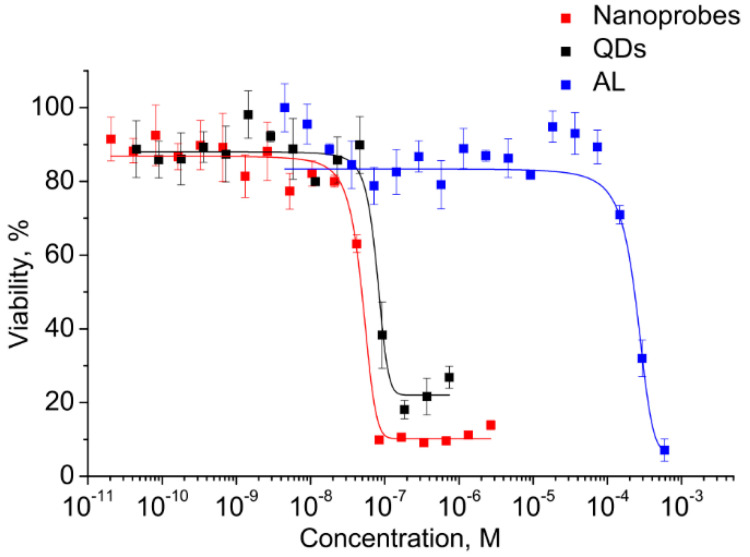
Cell viability in the presence of the conjugate and its components.

Before the functional evaluation of QDs, the half maximal inhibitory concentrations (IC_50_) of both components and the electrostatic conjugates were determined for estimating the differences in cytotoxicity and to estimate the operating QD concentration range. This analysis was performed in vitro using the cell viability assay (MTT assay) with monocytes. A human monocyte cell line was used because the cells have a high ability to uptake foreign objects, including nanoparticles and QDs, which also makes the cells a convenient model for investigation of the penetration of water-soluble QDs and biological imaging. The monocytes were incubated in a medium containing QDs, AL, and conjugates at concentrations ranging from 10^−3^ to 10^−11^ M. The monocyte viability was determined after 24 h of incubation. As shown in Figure 5, the viability of the cells was decreased with increasing concentration.

The graphically estimated IC50 values of AL, CdSe/ZnS QDs, and AL–QD nanoprobes were (2 ± 0.11) × 10^−4^, (5 ± 0.27) × 10^−8^, and (8 ± 0.15) × 10^−8^ M, respectively.

This has permitted us to identify the highest CdSe/ZnS QD concentration at which they can be regarded as reasonably safe, low-toxic agents for cell culture applications. The QDs are four orders of magnitude more toxic than AL. Therefore, the concentrations of QDs as the most toxic component were used to set the working concentration limits in the subsequent experiments on cells.

**Figure 6:**
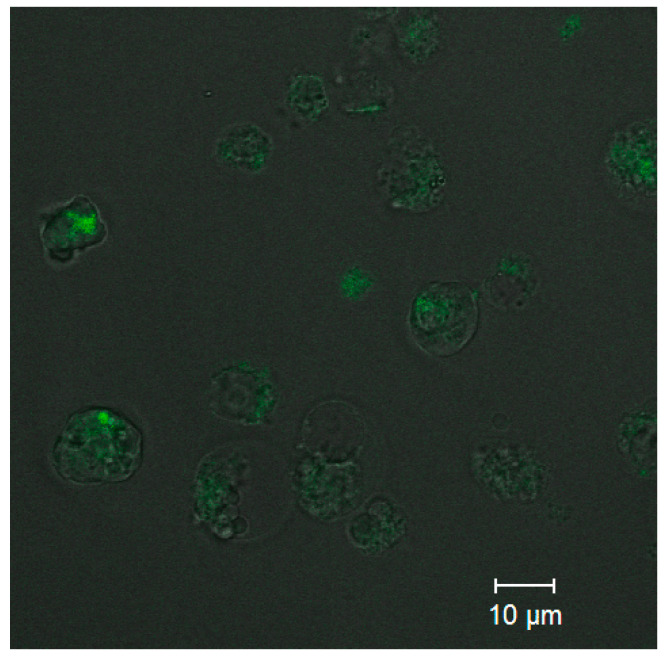
Confocal fluorescence microscopy images of the QDs in monocytes.

Analysis of the uptake of ultrasmall CdSe/ZnS QDs was performed to estimate the capacity of the PEGylated water-soluble QDs for penetrating into cells while retaining their PL capacity. Confocal fluorescence microscopy images (Figure 6) detected PL from the cells that was more intense than the background PL of the extracellular medium. The results showed that QDs at a concentration of 0.5 µM were detectable inside the monocytes after 10 min of incubation. As one can see from Figure 6, the cell state after CdSe/ZnS QD penetration is not good due to the high toxicity of CdSe/ZnS QDs. This is why the optimal conjugate concentration to be used for cell imaging should be carefully selected in each particular case. In addition, the confocal fluorescence brightness and the concentration of CdSe/ZnS QDs in cells could not be determined quantitatively at this stage of research, and more sophisticated approaches will be used to solve this problem in the future.

## 4. Conclusions

Homogeneous, defect-free ultrasmall CdSe/ZnS QDs have been synthesized, purified, solubilized in aqueous solutions, and conjugated with acridine derivatives, potential anticancer agents capable of effectively stabilizing the telomeric G-quadruplex sequences of human DNA. Ultrasmall CdSe cores 1.8 nm in diameter were obtained by means of optimized colloidal hot injection synthesis modified with the fast termination of QD growth using liquid nitrogen. The average diameter of the CdSe/ZnS QDs estimated using HRTEM has been determined to be 4.2 nm, and water-solubilized QDs have an average hydrodynamic diameter less than 12 nm and excellent colloidal stability, which makes them promising for intracellular studies. The highest concentration at which the synthesized QDs and their conjugates can be regarded as reasonably low-toxic agents for cell culture applications has been determined, and the uptake of the AL–QD conjugates by monocytes has been analyzed. The results have shown that the QDs at a concentration of 0.5 µM are detectable inside monocytes after 10 min of incubation. The method of compact nanoprobe engineering developed here is versatile because the ligand-coated QD surface allows their conjugation not only with acridine derivatives, but also with other pharmacological agents.

## Figures and Tables

**Table 1:** Main optical characteristics of CdSe/ZnS QDs with cores purified using different procedures.

Sample #	Sample	Absorbance Peak, nm/PL peak, nm/FWHM, nm	PL QY, %
1	CdSe/ZnS	-/498/73	63 (relative to Coumarin 102)
2	CdSe/ZnS with GPC-purified cores	447/479/46	68 (relative to Coumarin 102)

## Data Availability

All data generated and/or analyzed during this study are included in this article.

## Supplementary Materials

The following are available online at https://www.mdpi.com/article/10.3390/nano11092160/s1, Figure S1: Absorbance and photoluminescence spectra of ~1.8 nm core CdSe nanocrystals, Figure S2: TEM and HRTEM images of CdSe/ZnS QDs. Panel a–TEM image; panel b–HRTEM image.
