# Supplementary material for: Conjugates of Ultrasmall Quantum Dots and Acridine Derivatives as Prospective Nanoprobes for Intracellular Investigations
Source: Nanomaterials (Basel). 2021 Aug 24;11(9):2160. doi: 10.3390/nano11092160 (PMC8471518; doi:10.3390/nano11092160)
Supplement: Supplementary file 1 [file nanomaterials-11-02160-s001.zip › nanomaterials-1314474-supplementary.pdf]

*nanomaterials*

Supplementary Materials

# Conjugates of Ultrasmall Quantum Dots and Acridine Derivatives as Prospective Nanoprobes for Intracellular Investigations

**Pavel Linkov [1,2], Pavel Samokhvalov [1], Maria Baryshnikova [1,3], Marie Laronze-Cochard [4], Janos Sapi [4], Alexander Karaulov [5] and Igor Nabiev [1,2,5,*]**

1 Laboratory of Nano-Bioengineering, National Research Nuclear University MEPhI (Moscow Engineering Physics Institute), 31 Kashirskoe Highway, 115409 Moscow, Russia; palinkov@mephi.ru (P.L.); p.samokhvalov@gmail.com (P.S.); ma_ba@mail.ru (M.B.)
2 Laboratoire de Recherche en Nanosciences, LRN-EA4682, Université de Reims Champagne-Ardenne, 51 Rue Cognacq Jay, 51100 Reims, France ; igor.nabiev@univ-reims.fr
3 Laboratory of Experimental Diagnostics and Biotherapy of Cancer, N.N. Blokhin Russian Cancer Research Center, 24 Kashirskoe Highway, 115478 Moscow, Russia
4 Institut de Chimie Moléculaire de Reims, Université de Reims Champagne-Ardenne, 51 Rue Cognacq Jay, 51100 Reims, France ; marie.cochard@univ-reims.fr (M.L.-C.); janos.sapi@univ-reims.fr (J.S.)
5 Laboratory of Immunopathology, Department of Clinical Immunology and Allergology Sechenov First Moscow State Medical University (Sechenov University), 8-2 Trubetskaya Str., 119991 Moscow, Russia; drkaraulov@mail.ru (A.K.)
* Correspondence: igor.nabiev@univ-reims.fr

**Citation:** Linkov, P.; Samokhvalov, P.; Baryshnikova, M.; Laronze-Cochard, M.; Sapi, J.; Karaulov, A.; Nabiev, I. Conjugates of Ultrasmall Quantum Dots and Acridine Derivatives as Prospective Nanoprobes for Intracellular Investigations. *Nanomaterials* **2021**, *11*, 2160. https://doi.org/10.3390/nano11092160

Academic Editor: Zili Sideratou

Received: 10 July 2021
Accepted: 20 August 2021
Published: 24 August 2021

**Publisher's Note:** MDPI stays neutral with regard to jurisdictional claims in published maps and institutional affiliations.

**Copyright:** © 2021 by the authors. Submitted for possible open access publication under the terms and conditions of the Creative Commons Attribution (CC BY) license (http://creativecommons.org/licenses/by/4.0/).

## Figures

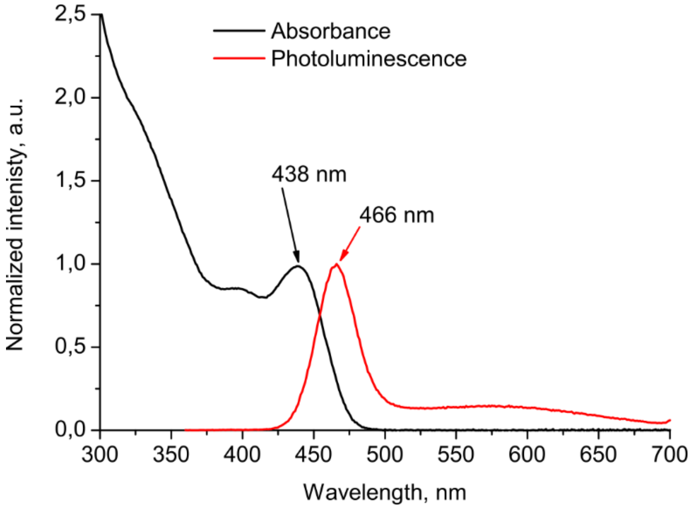


**Figure S1.** Absorbance and photoluminescence spectra of ~1.8 nm core CdSe nanocrystals.

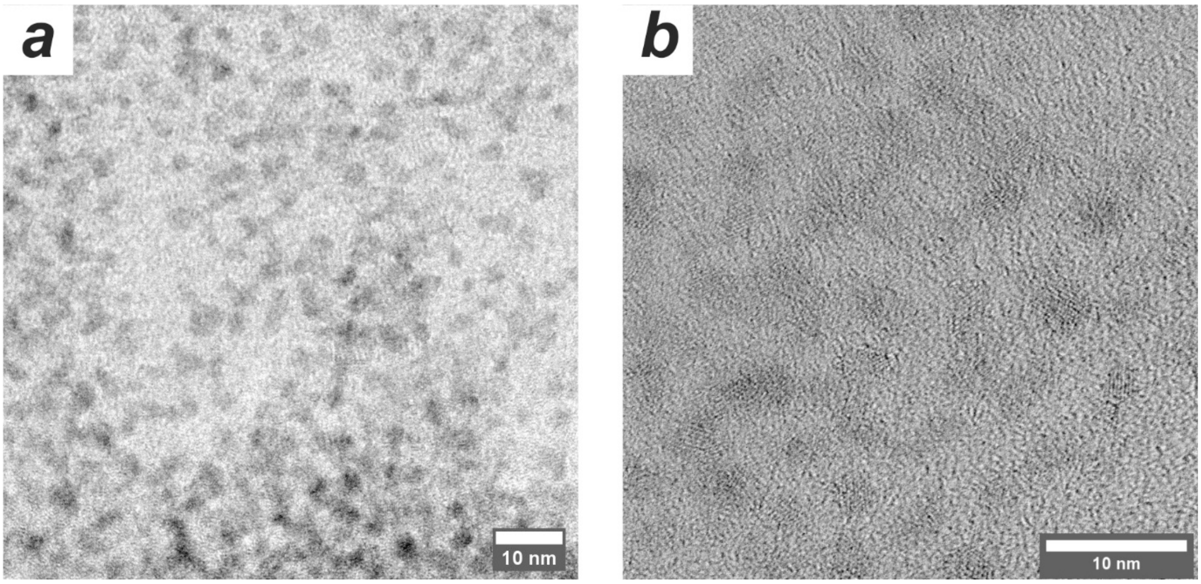


**Figure S2.** TEM and HRTEM images of CdSe/ZnS QDs. Panel a – TEM image; panel b – HRTEM image.
